# Acute and long‐term effect of specific and non‐specific exercises in patients with chronic neck pain: A protocol for a randomized controlled trial

**DOI:** 10.1113/EP091907

**Published:** 2024-10-19

**Authors:** Giovanna Laura Neves Antonio Gaban, Henrik Bjarke Vægter, Maria Ramela Schalch Vivaldini, Camila Nepomuceno Broisler, Giovanna Silva Nunes, Luiz Fernando Approbato Selistre

**Affiliations:** ^1^ Department of Physical Therapy Federal University of São Carlos (UFSCar) São Carlos São Paulo Brazil; ^2^ Pain Research Group, Pain Center Odense University Hospital Odense Denmark; ^3^ Faculty of Health Sciences, Department of Clinical Research University of Southern Denmark Odense Denmark

**Keywords:** chronic pain, exercise‐induced hypoalgesia, exercise therapy, neck pain

## Abstract

Exercise therapy is the most common approach for people with chronic neck pain (CNP). Although well‐established, it remains unknown which type of exercise is the best for treating this condition. Moreover, pain processing can play a role in the persistence of pain and in the response to interventions. Thus, the aim of this randomized controlled trial is to compare the acute and long‐term effects of two exercise protocols (specific and non‐specific) on pain and pain processing in individuals with CNP. One hundred and ten participants aged between 18 and 65 years who have had non‐specific neck pain for more than 3 months will be recruited. They will be randomized and allocated into two groups (specific exercises and non‐specific exercises) and both groups will perform an exercise programme twice a week for 8 weeks. Both programmes are divided into two progressive and individualized phases. The primary outcomes are change in pain intensity after 8 weeks of exercise and exercise‐induced hypoalgesia, and secondary outcomes are pressure pain threshold, temporal summation of pain, conditioned pain modulation, the Neck Disability Index, the Baecke Physical Activity Questionnaire, and the Global Perception of Change Scale. Outcomes will be assessed at baseline, after 8 weeks of intervention, and at 6‐month follow‐up.

## Abstract (highlights)

- **What is the central question of this study?**
  Chronic neck pain is the second‐most disabling musculoskeletal condition: how do the acute and long‐term effects of specific and non‐specific exercises for chronic neck pain compare?
- **What is the importance of this work?**
  This is the first randomized controlled trial to compare the effects of acute and long‐term effects of specific and non‐specific exercises on pain intensity and pain processing in chronic neck pain.

## 1 INTRODUCTION

Chronic neck pain (CNP) is the health condition that causes the fifth most disability in the world, and among musculoskeletal conditions, it is second only to low back pain (Vos et al., 2017). It affects 30–50% of the general population, mainly women, with a higher prevalence in middle age (Hogg‐Johnson et al., 2009). It is considered an important public health problem with great economic impact, impairment of productivity and absenteeism (Fandim et al., 2020; Hogg‐Johnson et al., 2009). Approximately, 50% of individuals who experience neck pain have had pain and disability for over a year, demonstrating poor recovery from the condition (Hush et al., 2011). The condition can be predicted by high initial levels of pain and disability, negative psychological factors, and a lower pressure pain threshold (PPT) (Fandim et al., 2020; Walton et al., 2009). In addition, individuals with CNP may have altered pain processing, measured by quantitative sensory testing (QST), and further investigations are needed since it can predict worse outcomes (Xie et al., 2020).

Currently, the literature recommends therapeutic exercises among treatment approaches for this population (Fandim et al., 2020; Gross et al., 2015). For chronic conditions, therapeutic exercises have proven to be an accessible and cost‐effective therapeutic modality (Boldt et al., 2014; Gross et al., 2015), although there is no consensus about the best type and dose of exercise (Blanpied et al., 2017; Villanueva‐Ruiz et al., 2022). Specific exercise (SE), described as exercising the painful area (Dueñas et al., 2021), is the most common type of exercise used in CNP, although it seems not to decrease local and remote pain sensitivity after a single bout of exercise (Naugle et al., 2012) – a phenomenon commonly known as exercise‐induced hypoalgesia (EIH; Koltyn et al., 2014). This hyperalgesia after exercise can be a major barrier to patient adherence to treatment, leading to physical inactivity and consequently worsening pain and disability (Jack et al., 2010).

On the other hand, non‐specific exercise (NSE), described as exercising the non‐painful area (Dueñas et al., 2021; Vaegter & Jones, 2020), seems to activate endogenous analgesic pathways (Naugle et al., 2012) and produce more EIH effects. It can be found in other chronic conditions such as knee osteoarthritis (Burrows et al., 2014) and shoulder myalgia (Lannersten & Kosek, 2010); however, in CNP, the EIH effect of NSE is not well established. In the last systematic review and meta‐analysis on this topic, Senarath et al. (2022) concluded that the evidence on EIH is conflicting and limited, caused by low methodological quality (Senarath et al., 2022; Vaegter & Jones, 2020). Besides this, SE cannot be considered superior to NSE in pain intensity and pain processing in CNP due to the small effect sizes and high risk of bias (Bontinck et al., 2024; Villanueva‐Ruiz et al., 2022), whether in acute or long‐term interventions (Hansen et al., 2022). It is also important to emphasize that the assessment of pain processing using QST, a method widely employed for this purpose (Xie et al., 2020), has not been well investigated in the area.

The primary aim of this randomized controlled trial (RCT) is to investigate if 8 weeks of NSE is superior in improving pain and EIH compared to SE in CNP. In addition, pain processing evaluated by QST will be compared between groups after the 8‐week intervention and at 6‐month follow‐up.

## 2 METHODS

### 2.1 Ethical approval

The study protocol has been approved by the Human Ethics Committee of the Federal University of São Carlos (UFSCar) under the following registration number: 67259123.0.0000.5504. It adheres to the guidelines set forth in Resolution 510/16 of the National Health Council concerning research involving human subjects. Informed consent will be obtained for all participants. The exercises described have minimal risk of adverse reactions, take place according to the usual clinical procedures, and are used only for people who speak and understand Brazilian Portuguese so that there are no language uncertainties in connection with the information provided. No placebo treatment is provided. For future inquiries regarding the data generated from this study, interested parties may contact the corresponding author, L.F.A.S. with a reasonable request.

### 2.2 Monitoring and auditing

This study does not have a formal data monitoring committee and no trial audit is planned as this trial does not investigate drugs or products. Adverse reactions will be discussed by all authors.

### 2.3 Study design

This study protocol describes the design of a single‐blind RCT, developed in accordance with the recommendations of SPIRIT (Standard Protocol Items: Recommendations for Interventions Trials) (Chan et al., 2013), CONSORT (Consolidated Standards of Reporting Trials) (Moher et al., 2001) and TIDieR (Template for Intervention Description and Replication) (Hoffmann et al., 2014). This study was registered in the Brazilian Clinical Trials Registry (RBR‐337hq7s)—https://ensaiosclinicos.gov.br/rg/RBR‐337hq7s.

### 2.4 Participants

Participants in this trial are women and men with chronic non‐specific neck pain.

#### 2.4.1 Eligibility criteria

Participants between 18 and 65 years (Bernal‐Utrera et al., 2020), who speak and understand Brazilian Portuguese, experiencing non‐specific CNP (pain in the area between the nuchal line and the spinous process of the first thoracic vertebra without having a known cause; Fandim et al., 2020) for at least 3 months (Gattie et al., 2021), with pain at rest and/or a neck active movement score of ≥3 on a numeric pain rating scale (NPRS) (Rampazo et al., 2021) and ≥10 points (20%) on the Neck Disability Index (NDI) (Daher et al., 2020; Gattie et al., 2021) will be included. Participants may have other pain complaints as long as neck pain is predominant.

#### 2.4.2 Exclusion criteria

Signs of radiculopathy will be verified by the Upper Limb Tension test, the Spurling test, the Traction/Distraction test (Bier et al., 2018), a history of radicular pain worse in the arm than in the neck, and the presence of paresthesia or numbness and/or weakness and/or altered reflex (Lam et al., 2022). Whiplash‐associated disorders, cervicogenic headache, fibromyalgia, pregnancy, history of trauma, cervical fractures or surgeries, inflammatory rheumatic diseases related to the cervical region, neurological disorders, tumors, and/or medical contraindication to physical exercise (Bernal‐Utrera et al., 2020; Daher et al., 2020; Gattie et al., 2021; Rampazo et al., 2021) will be also excluded. Individuals who received physiotherapy or cervical infiltration in the last 3 months, initiated some physical activity in the last 2 weeks, or used analgesics, anti‐inflammatory drugs or muscle relaxants acutely within 24 h before the evaluation will also be excluded (Rampazo et al., 2021, 2020).

### 2.5 Recruitment procedure

Recruitment will take place through the dissemination of advertisements in social media networks, local newspapers and television. An online form will assess eligibility criteria, followed by a face‐to‐face visit for confirmation and informed consent. When eligible participants consent to participate, they will be randomized to one of the two exercise groups.

### 2.6 Randomization and blinding

Electronic randomization will be performed (https://www.randomizer.org/) by a researcher not involved in the assessments or intervention. The allocation will be hidden in opaque, sealed and consecutively numbered envelopes stored securely. The therapist will open the sealed envelope in front of the participant before starting the intervention. Due to the nature of interventions (exercises), it is not possible to blind the physiotherapist. However, the evaluator will be blinded to the treatment group, and physiotherapists involved in treatment will be blinded to evaluation results. The effectiveness of blinding will be assessed after the 6‐month follow‐up. The researcher will record responses indicating whether she thinks the participant received SE, NSE, or if she does not know how to evaluate blinding effectiveness (Coppieters et al., 2021).

### 2.7 Exercise interventions

Both groups will follow a home‐based exercise programme comprising stretching, mobility and strengthening exercises (Price et al., 2021) lasting 24 exercise sessions over 8 weeks, with a weekly frequency of three sessions, each lasting approximately 30 min, as recommended in guidelines (Booth et al., 2017; Ferro Moura Franco et al., 2021). The exercise programmes will be explained, and videos previously recorded with all the instructions needed will be provided.

Both groups will perform a previous effective protocol for decreasing pain intensity. The SE group will perform a protocol adapted from Celenay et al. (2016), while the NSE group will perform a protocol targeting the main muscle groups of the lower limbs (Ferreira et al., 2007) (Table 1). The exercise dose will be individualized based on recommendations by Price et al. (2021), utilizing body weight or elastic bands (Andersen et al., 2011; Celenay et al., 2016). Throughout the intervention, the training load will progressively increase, adhering to the principle of progressive overload (Progression Models in Resistance Training for Healthy Adults, 2009). Participants are expected to advance the exercise load when they surpass the specified repetitions or classify the Perceived Effort with a score equal to or less than 6 on the BORG Scale (Iversen et al., 2018; Morishita et al., 2018). In addition, there will be two phases of progression. Following the intervention, participants will be encouraged to continue exercising using videos tailored to their randomized group (O'Riordan et al., 2014; Suni et al., 2017).

**TABLE 1:** Intervention protocol exercises for both groups (specific and non‐specific).

Type of exercise	Exercise	Dose
Specific exercise group		
Phase 1 (weeks 1–4)		
Mobility/flexibility	Thoracic and cervical mobility on four supports	Two sets of 8–10 repetitions each
Strengthening	Cervical flexion in supine position	Three sets of 8–10 repetitions each
	Cervical extension in prone position	
	Scapular retraction with arms extended in prone position	
	Lateral shoulder raise	
Mobility/flexibility	Stretching for cervical extensors	One set lasting 30 s
	Stretching for cervical tilters and rotators	One set lasting 30 s

Phase 2 (weeks 5–8)		
Mobility/flexibility	Thoracic rotation in lateral decubitus	Two sets of 8–10 repetitions each
Strengthening	Cervical flexion with craniocervical flexion in supine position	Three sets of 8–10 repetitions each
	Cervical extension with elastic band in prone position	
	Scapular retraction and horizontal shoulder abduction with elastic band while standing	
	Shoulder abduction and elbow extension (Arnold shoulder press)	
Mobility/flexibility	Stretching for cervical extensors	One set lasting 30 s
	Stretching for cervical tilters and rotators	

Non‐specific exercise group		
Phase 1 (weeks 1–4)		
Mobility/flexibility	Flexion of hips and knees in supine position	Two sets of 8–10 repetitions each
Strengthening	Wall squat	Three sets of 8–10 repetitions each
	Hip abduction with elastic band seated	
	Hip raise in supine position (bridge exercise)	
	Extended leg raise in supine position	
Mobility/flexibility	Stretching for hip flexors	One set with 30 s of duration
	Stretching for hip extensors	One set with 30 s of duration

Phase 2 (weeks 5–8)		
Mobility/flexibility	Half‐kneeling hip mobility	Two sets of 8–10 repetitions each
Strengthening	Lunge	Three sets of 8–10 repetitions each
	Squat with hip abduction with elastic band (side lunge)	
	Unilateral hip raise in supine position (unilateral bridge)	
	Hip and knee flexion with elastic band while standing	
Mobility/flexibility	Stretching for hip flexors	One set lasting 30 s
	Stretching for hip extensors	

### 2.8 Data collection procedure

Data collection, physical examination and QST will be conducted by a blinded evaluator.

### 2.9 Outcomes

**FIGURE 1:**
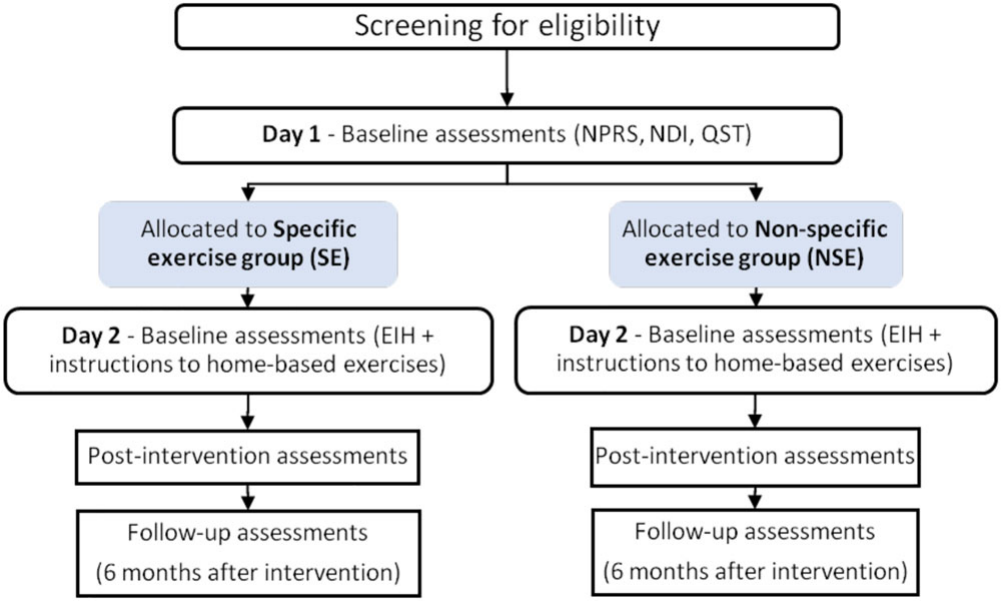
Flowchart summarizing experimental procedures.

Outcome assessments will be performed at baseline, post‐intervention (8 weeks), and 6‐month follow‐up (Figure 1). For all assessments, participants will be instructed to avoid alcoholic and caffeinated beverages 24 h before the scheduled date (Marcuzzi et al., 2017). The evaluation room will be maintained at an approximate temperature of 23°C and without noise (Marcuzzi et al., 2017).

#### 2.9.1 Primary outcomes

##### Pain intensity at rest and on movement

Pain intensity will be evaluated through the numerical pain rating scale (NPRS) (Ferreira‐Valente et al., 2011). This scale ranges from 0 (no pain) to 10 (worst possible pain) and will be questioned at rest (Celenay et al., 2016) and different active cervical movements (flexion, extension, inclinations and rotations) (Rampazo et al., 2020). The highest value will be used for analysis. The minimal clinically important difference (MCID) is 2.5 points on a scale of 0–10 (Pool et al., 2007).

##### Exercise‐induced hypoalgesia

**FIGURE 2:**
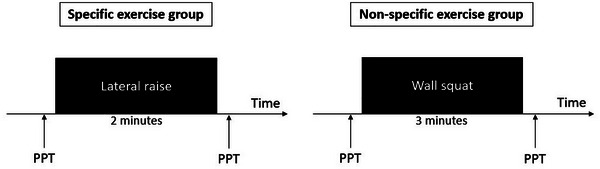
Illustration of the experimental procedure for assessing the acute effect. Pressure pain threshold (PPT) will be measured at points *X*, *Y*, and *Z* before and after the randomized exercise. Participants assigned to the specific exercise group will perform a lateral raise for 2 min or until fatigue. Participants assigned to the non‐specific exercise group will perform an isometric wall squat for 3 min or until fatigue.

The blinded evaluator will measure the PPT both before and immediately after the exercise (Figure 2). EIH will be calculated as the difference between PPT immediately after the exercise protocol and PPT before exercise at the points of left upper trapezius, left quadriceps femoris, and right low back (Hansen et al., 2022; Christensen et al., 2022; Vaegter et al., 2021) (Figure 2). A positive value reflects EIH, and a higher value indicates more effective EIH (Xie et al., 2021). Participants will perform an exercise to evaluate EIH based on the group to which they were randomized (Table 2).

**TABLE 2:** Description of the exercises that will be used to verify the acute effect of exercise through exercise‐induced hypoalgesia.

	Specific exercise group	Non‐specific exercise group
Exercise	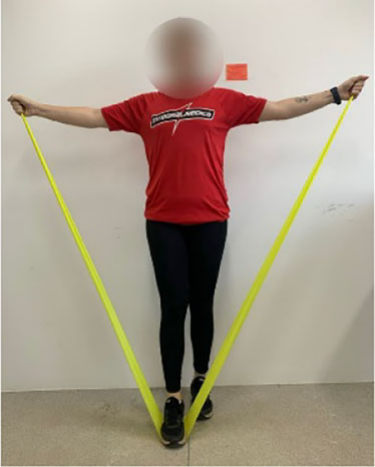	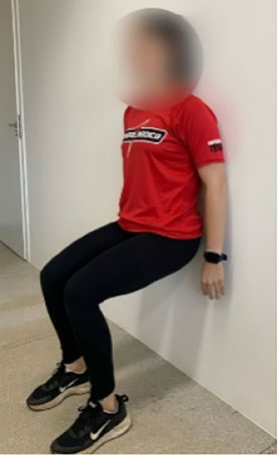
	Lateral raise	Isometric wall squat
Duration	2 min or until fatigue	3 min or until fatigue
Instructions	The lateral raise will be performed with a light‐intensity elastic band. The participant will be instructed to perform the exercise in a controlled manner, taking approximately 2 s for both the raising and lowering phases (Andersen et al., 2011; Hansen et al., 2020). The participant will be guided to raise the arm to approximately 90° with the elbows slightly flexed. The exercise will be continued for the specified duration or until fatigue (Hansen et al., 2020).	The wall squat will be performed with the participant positioned against a wall, with feet parallel and hands at the side of the body. The knee flexion angle, measured with a goniometer aligned on the lateral epicondyle of the femur, should reach approximately 100° (Vaegter et al., 2019). Once positioned, participants will be prompted to sustain the crouched posture for the designated time or until muscle fatigue sets in. Pain intensity, measured by the Numeric Pain Rating Scale (NPRS), and perceived exertion will be recorded at the conclusion of the exercise (Smith et al., 2017).

#### 2.9.2 Secondary outcomes

##### Pressure pain threshold

PPT will be measured using a digital pressure algometer (Algomed, Computerized Pressure Algometer, Medoc, Israel) positioned perpendicular to the skin with a pressure rate of approximately 40 kPa/s (Rampazo et al., 2020). A familiarization with an unrelated region (hand) will precede data collection (Andersen et al., 2012; Xie et al., 2021). All points are described in Table 3. Points will be randomized, and participants, with closed eyes, will press a button on the algometer with the hand contralateral to the evaluated limb and will be instructed to press it when the pressure stimulus becomes a clear sensation of pain. Three measurements will be taken at each point with a 30‐s interval between them (Walton et al., 2014), and the average will be used for analysis (Lluch et al., 2014). PPT in neck pain has good reliability with ICC = 0.89 for intrarater and 0.83 on test–retest, with a minimum detectable change (MDC) of 155.73 kPa and good responsiveness with area under the curve (AUC) = 0.76 (Walton et al., 2014).

**TABLE 3:** Description of the points that will be used in the pressure pain threshold.

Point	Description	Position
Bilaterally C2	2 cm laterally from the spinous processes of C2	Ventral decubitus with the face resting on the stretcher hole to keep the cervical spine neutral and arms along the body (Rampazo et al., 2021, 2020)
Bilaterally upper trapezius	Midway between the spinous process of C7 and the lateral edge of the acromion (Celenay et al., 2016; Rampazo et al., 2021)	Sitting on a chair with feet flat on the floor and trunk resting on the back of the chair (Rampazo et al., 2021, 2020)
Right anterior tibialis	Middle third of the right tibialis anterior muscle (Hansen et al., 2020; Rampazo et al., 2021)	Dorsal decubitus with knees bent and feet resting on the stretcher (Walton et al., 2011)
Right low back	5 cm laterally from the spinous process of L3 (van Oosterwijck et al., 2012)	Prone position with arms along the body (van Oosterwijck et al., 2012)
Left quadriceps femoris muscle	20 cm above the center of the left patella (Hansen et al., 2020; Vaegter et al., 2014).	Supine position with a pillow under the knee (Hansen et al., 2020)

##### Temporal summation of pain

Temporal summation of pain (TSP) will be evaluated at the right upper trapezius. Ten pressure stimuli will be applied at the previously determined pressure value, with a 1 s interval between them. Participants will quantify pain intensity for the first, fifth and tenth stimuli using the NPRS (Corrêa et al., 2016). The difference between the tenth pain intensity stimuli and the first will be used for analysis. TSP has demonstrated good reliability with ICC = 0.75 (Cathcart et al., 2009).

##### Conditioned pain modulation

Conditioned pain modulation (CPM) will be evaluated using a cold conditioning stimulus test, with the PPT at the left upper trapezius and right quadriceps femoris points as the test stimulus. Participants will be instructed to immerse their right foot (up to the ankle) in a bucket of ice water at 4°C for 1 min (Shahidi & Maluf, 2017). Before removing the foot from the water, participants will rate the pain intensity in the foot based on the NPRS (Vaegter et al., 2014; Yarnitsky et al., 2015). After 1 min, they will remove their foot from the water to avoid distraction bias, and PPT on the upper trapezius and quadriceps femoris will be measured again (Valencia et al., 2013; Yarnitsky et al., 2015). The values obtained before and after immersion will be subtracted (PPT after the conditioning stimulus minus PPT before the conditioning stimulus) to evaluate the effectiveness of CPM. A positive value reflects CPM, and the higher the value, the more efficient the endogenous pain inhibition (Xie et al., 2021). Data will be reported in absolute difference, as recommended in the literature (Kennedy et al., 2016).

##### Neck Disability Index

The NDI will be used to assess disability of patients with neck pain. The translated and validated version for Brazilian Portuguese (Cook et al., 2006) demonstrated good internal consistency and reliability (Cook et al., 2006). The NDI consists of 10 questions rated from 0 (no disability) to 5 (greater disability). The total score is expressed as a percentage of the maximum possible score according to the number of questions answered (Chung & Jeong, 2018). The MDC is 10.5 points (Pool et al., 2007) while the MCID is 7 points, both on a scale of 0–50 (Abbott & Schmitt, 2014).

##### Global Rating of Change

The Global Rating of Change (GROC) will be used to assess the global perception of change after the 8‐week intervention and at the 6‐month follow‐up. The GROC is a 15‐point scale ranging from −7 (a very great deal worse) to 0 (about the same) to +7 (a very great deal better). It will be used to quantify a patient's improvement with treatment (Kamper et al., 2009). It has excellent reliability (ICC between 0.80 and 0.92) on test–retest (Bobos et al., 2019) and is considered a valid reference standard for identifying clinically important changes (Cleland et al., 2007). A score of +4 will indicate positive improvement (Cleland et al., 2007), with the MCID set at a 3‐point difference from the baseline (Daher et al., 2020). This scale will be collected in relation to cervical symptoms.

##### The Baecke Physical Activity Questionnaire

The Baecke Physical Activity Questionnaire (BPAQ) will assess the level of physical activity. This self‐reported questionnaire considers three indices of habitual physical activity (work, sports and leisure activities). Comprising 16 questions, the total score is the sum of all the questions (each rated from 0 to 5 points). A higher final score indicates a higher level of physical activity. It shows good reliability (Baecke et al., 1982; Silva et al., 2020) and is validated with the gold standard for the level of physical activity using the Doubly Labeled Water (Philippaerts et al., 1999).

The following will be also collected:

Exercise adherence: Participants will be given an adherence diary to fill in every week and also text messages will be sent to ask them about the exercise and adherence. Monthly calls or text messages, according to the participant's preference, will be made, to stay in touch with participants during the follow‐up period.
Adverse events: In the weekly contact with the participant, questions regarding adverse events (such as pain, headache, dizziness or other symptoms that may occur) will be made and the answers will be registered.

### 2.10 Sample size

The sample size was calculated using G*Power 3.1.9.7 based on analysis of variance (ANOVA) repeated‐measures, within–between interactions (two groups and three measurements). Thus, this study is designed to detect a small effect size (eta square of 0.02; Daher et al., 2020) in pain intensity. To detect this difference, 41 participants in each group are needed (assuming a power of 0.80, α level of 0.05 and critical *F* = 3.052). Considering a 15% dropout, the study will include 94 participants, with 47 in each group.

### 2.11 Statistical analysis

Patient characteristics at baseline will be reported with descriptive statistics as means and standard deviation (SD), median and interquartile range (IQR), or numbers and percentages as appropriate. Normality will be visually assessed using histograms and the Shapiro–Wilk test.

Using intention‐to‐treat analysis, treatment effects, differences between groups and confidence intervals (95%) will be calculated through repeated‐measures ANOVA, examining the interaction between treatment groups (SE and NSE) over time (baseline, post‐intervention, and 6‐month follow‐up). If the ANOVA results are statistically significant, univariate analysis will be conducted using the Tukey *post hoc* test, with Bonferroni adjustment for multiple comparisons. Effect size will be calculated using Cohen's *d*, categorized as small (0.02), medium (0.05), and large (0.08) (Cohen, 1988). IBM SPSS Statistics (Version 21.0; IBM Corp., Armonk, NY, USA) will be used for all analyses.

#### 2.11.1 Dissemination of results

Results will be published in peer‐reviewed scientific journals, presented at conferences, communicated to patient organizations, and reported in social media.

## 3 DISCUSSION

### 3.1 Impact and significance of study

This is the first randomized trial comparing the acute and long‐term effects of SE and NSE on pain and pain processing in CNP. Despite the lack of consensus on the optimal type and dose of exercise, it is recommended as a first‐line treatment for this population (Blanpied et al., 2017; Fandim et al., 2020; Villanueva‐Ruiz et al., 2022; Wilhelm et al., 2020). In addition, pain processing significantly influences CNP persistence and prognosis (Georgopoulos et al., 2019; Xie et al., 2020) contributing to the poor response to conventional treatment (Jull et al., 2007; Xie et al., 2020). The results will provide important knowledge about the effects of SE and NSE and the findings can influence future care. The study's results will provide valuable knowledge supporting clinical practice by assisting physiotherapists in prescribing therapeutic exercises to enhance effects after exercise. This, in turn, could improve adherence to long‐term treatments and deepen our understanding of the effects of both SE and NSE in individuals with CNP.

### 3.2 Strengths and limitations

One notable strength of this study is the robust and rigorous methodology. It is a randomized trial, prospectively registered, with the blinding of the evaluator, and the sample size was determined to ensure sufficient statistical power for accurately detecting significant between‐group differences in the primary outcome. Additionally, the exercise programmes are designed to be simple for home use, considering that the majority of the individuals with CNP are economically active and may have limited time for daily treatment. Besides, the protocol's simplicity and detailed description – including exercise form, intensity, frequency and progressions – align with expert recommendations, addressing a gap in the literature (Silva et al., 2019).

The study has some limitations: the absence of a control group, such as a waiting list or placebo intervention, could potentially confound the results, although therapeutic exercise is established as superior to a control group (Andersen et al., 2014; Garzonio et al., 2022; Wilhelm et al., 2020). Adherence should be interpreted cautiously as the intervention is conducted at home. Lastly, due to the nature of the intervention (exercises), participants and physiotherapists will not be blinded.

## AUTHOR CONTRIBUTIONS

Giovanna L. N. A. Gaban, Henrik B. Vægter, and Luiz F. A. Selistre studied the concept or design of the work. Giovanna L. N. A. Gaban, Henrik B. Vægter, Maria R. S. Vivaldini, Camila N. Broisler, Giovanna S. Nunes, and Luiz F. A. Selistre had drafted the article or revised it critically for important intellectual content. Giovanna L. N. A. Gaban, Henrik B. Vægter, Maria R. S. Vivaldini, Camila N. Broisler, Giovanna S. Nunes, and Luiz F. A. Selistre gave final approval for the version to be published. All authors approved the final version of the manuscript and agree to be accountable for all aspects of the work in ensuring that questions related to the accuracy or integrity of any part of the work are appropriately investigated and resolved. All persons designated as authors qualify for authorship, and all those who qualify for authorship are listed.

## CONFLICT OF INTEREST

None declared.
